# The complete chloroplast genome of *Spiraea mongolica* Maxim

**DOI:** 10.1080/23802359.2021.1926351

**Published:** 2021-05-12

**Authors:** Yanjun Ma, Yupeng Guo, Ye Zhu, Qiujuan Liu, Lina Gao, Wenjing Yang, Xie Jun, Rui Ma

**Affiliations:** a College of Forestry, Gansu Agricultural University, Lanzhou, P. R. China; b Qinghai Provincial Key Laboratory of High value Utilization of Characteristic Economic Plants, College of Ecological Environment and Resources, Qinghai Nationalities University, Xining, P. R. China; c Ningxia Forestry Institute, Yinchuan, P. R. China

**Keywords:** *Spirea mongolica* Maxim, chloroplast genome, phylogenetic analysis

## Abstract

The complete chloroplast genome of *Spirea mongolica* Maxim. was sequenced and assembled. It is a circular form of 155949 bp in length, which was separated into four distinct regions, a large single copy (LSC) of 84375 bp, a small single copy region (SSC) of 18894 bp, two inverted repeats (IR) of 26340 bp. After annotation, a total of 115 genes were predicted, of which, 70 encode proteins, 8 rRNA, 37 tRNA. The evolutionary history, inferred using Neighbour-Joining method, indicates that *S. mongolica* was grouped within Rosaceae, and comprised a clade with *Spirea blumei* G.Don, another species in Spirea, with 100% BS value.

*Spirea mongolica* Maxim, belonging to Rosaceae, is an alpine perennial shrub with little white flowers. It is always found in western China and some areas of Mongolia and Siberia. On the Qinghai–Tibet Plateau and adjacent highlands, it can survive at altitudes of 2,000–4,500 m. This plant is often used as folk Tibetan medicine and Mongolia medicine to treat swelling and ulcer on the body surface, trauma, ascites, pulmonary congestion, uterine bleeding and other diseases. Some literatures even reported this plant contains some potential gradient, probably botulin and its derivatives, in anti-HIV and cancer therapy (Huanwen et al. 2017). Some papers about its screening of microsatellite markers, phylogeography and evolution were also found (Tiejuan and Yi-Zhi 2002, 2012; Gulzar et al. 2014, 2018). In this study, we reported its complete chloroplast genome.

Samples from Qilian mountains (36°34′37″N,101°48′27″E) in Qinghai province were collected. Voucher specimen (GAUF20200531SMNO001) was deposited in the Herbarium, College of Forestry, Gansu Agricultural University. A sample was used for chloroplast genome sequencing, performed on the Illumina NovaSeq platform (Nanjing Jisihuiyuan biotechnology Co. Ltd). The complete cp genome was assembled with the de novo assembler SPAdes (Bankevich et al. 2012). Gene annotation was performed via PGA (Qu et al. 2019).

The complete cp genome of *S. mongolica* (GenBank accession no.MT732945) has a typical quadripartite form of 155,949 bp in length, and composed of a large single copy region (LSC, 84375 bp), a small single copy region (SSC, 18894 bp), two inverted repeats (IR, 26340 bp). GC content of the genome is 36.74%. A total of 115 genes were predicted on this cp genome, of which, 70 encode proteins, 8 rRNA, 37 tRNA.

Phylogenetic analysis was performed based on complete cp genomes of *S. mongolica* and other 28 related species reported in Rosaceae, two species in Leguminosae as out-group. The sequences were aligned using MAFFT (Katoh et al. 2002) and trimAl was employed to remove ambiguously aligned sites (Capella-Gutierrez et al. 2009). The evolutionary history was inferred using Neighbour-Joining method in MEGA7.0 (Kumar et al. 2016). Bootstrap (BS) values were calculated from 1000 replicates (Figure 1). As expected, *S. mongolica* was grouped within Rosaceae, and comprised a clade with *Spirea blumei* G.Don, another species in *Spirea*, with 100% BS value. *The* complete cp genome of *S. mongolica* will be helpful for further studies on population genetics, taxonomy or resources protection.

**Figure 1.**
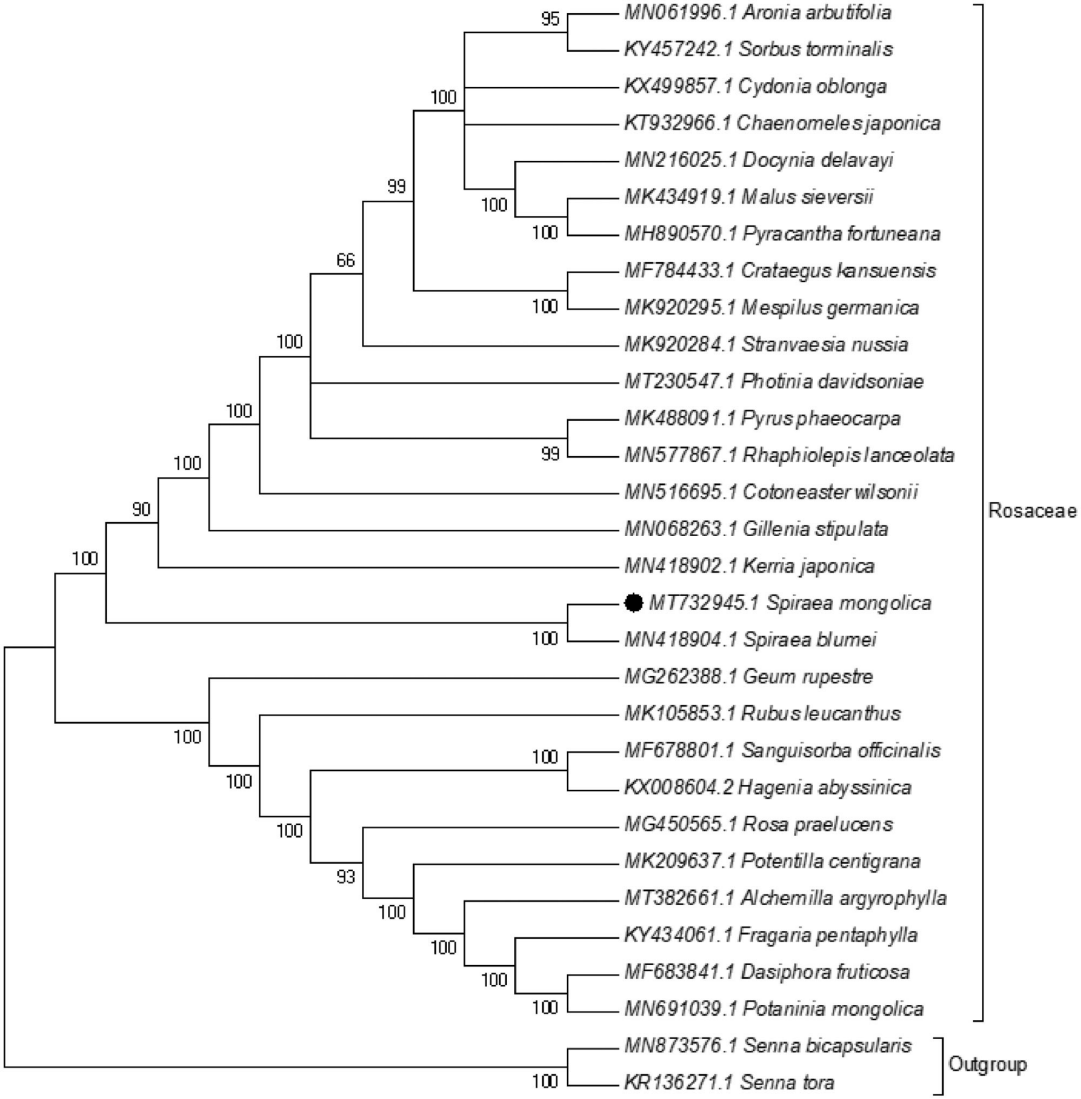
NJ phylogenetic tree based on 30 species chloroplast genomes was constructed using MEGA7.0. Numbers on each node are bootstrap from 1000 replicates.

## Data Availability

The genome sequence data that support the findings of this study are openly available in GenBank of NCBI at (https://www.ncbi.nlm.nih.gov/nuccore/MT732945.1) under the accession no. MT732945.1. The associated BioProject, SRA, and Bio-Sample numbers are PRJNA678470, SRR13062012, and SAMN16802410, respectively.
